# Systematic review: diet–gene interactions and the risk of colorectal cancer

**DOI:** 10.1111/apt.12180

**Published:** 2012-12-10

**Authors:** V Andersen, R Holst, U Vogel

**Affiliations:** 1 Medical Department, Hospital of Southern JutlandAabenraa, Denmark; 2 Institute of Regional Health Research, Faculty of Health Sciences, University of Southern DenmarkOdense, Denmark; 3 Medical Department, RHV ViborgViborg, Denmark; 4 National Research Centre for the Working EnvironmentCopenhagen, Denmark

## Abstract

**Background:**

Diet contributes significantly to colorectal cancer (CRC) aetiology and may be potentially modifiable.

**Aim:**

To review diet–gene interactions, aiming to further the understanding of the underlying biological pathways in CRC development.

**Methods:**

The PubMed and Medline were systematically searched for prospective studies in relation to diet, colorectal cancer and genetics.

**Results:**

In a meta-analysis, no interaction between NAT1 phenotypes and meat intake in relation to risk of CRC was found (*P*-value for interaction 0.95). We found a trend towards interaction between NAT2 phenotypes and meat intake in relation to risk of CRC. High meat intake was not associated with risk of CRC among carriers of the slow NAT2 phenotype, whereas *NAT*2 fast acetylators with high meat intake were at increased risk of CRC (OR = 1.25; 95% confidence interval (CI): 0.92–2.01) compared with slow acetylators with low meat intake (reference), *P*-value for interaction = 0.07. Low meat intake in the studied populations may influence the result. Interactions between meat, cruciferous vegetables, fibres, calcium, vitamins, and alcohol and *ABCB1*,*NFKB1*,*GSTM1*, *GSTT1*, *CCND1*, *VDR*, *MGTM*, *IL10* and *PPARG* are suggested.

**Conclusions:**

A number of interactions between genetic variation and diet are suggested, but the findings need replication in independent, prospective, and well-characterised cohorts before conclusions regarding the underlying biological mechanisms can be reached. When the above criteria are met, studies on diet–gene interactions may contribute valuable insight into the biological mechanisms underlying the role of various dietary items in colorectal carcinogenesis.

## Introduction

Colorectal cancer (CRC) constitutes the second most common cancer in the Western World^1^ and the prevalence is expected to increase due to demographic trends and adaption to westernised lifestyle in developing countries.^2^ Suspected or established risk factors include diet, obesity, physical inactivity, diabetes mellitus, smoking, family history of CRC, and inflammatory bowel disease.^1^ More than 50% of the aetiology has been attributed to diet and lifestyle^1^,^3^, and, may therefore be potentially avoidable by modification of these factors.^2007^

This article reviews diet–gene interactions to understand the underlying biological pathways by which diet affects colorectal carcinogenesis and to provide a basis for translating this knowledge into efficient preventive and treatment strategies.

## Identification of Diet–Gene Interactions

**Figure 1:**
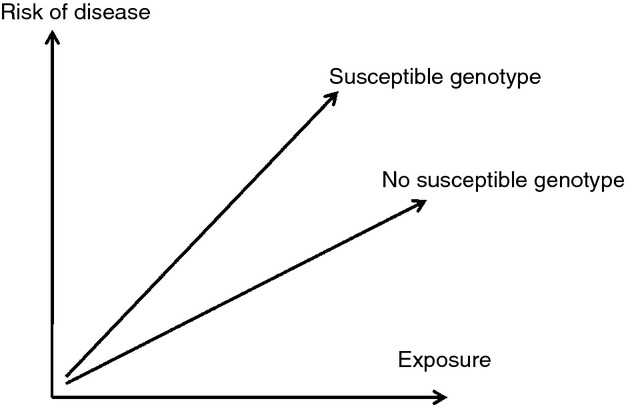
Individual genetic susceptibility may modify the effect of dietary components on colorectal carcinogenesis (see text).

Polymorphisms in low-penetrance genes may modify the risk conferred by environmental factors and the assessment of such gene–environmental interactions may be utilised for identification of biological pathways (Figure 1). The attributable risk in the population may be large when the variant allele frequency is high even if the associated increase or decrease in cancer risk is small. Many of these low-penetrance genes may be identified in the context of exposure and not as main effect.^5^,^6^, Therefore, the successful identification of gene–environmental interactions requires assessment of genetic polymorphisms in combination with accurate estimates of the environmental exposure under study.

## Materials and Methods

The PubMed and Medline were systematically searched for studies with the scope diet and risk of CRC (May 2012) using the following terms: diet, nutrients, colorectal cancer, colorectal neoplasm (epidemiology or etiology or genetics or prevention and control), genetic variant, polymorphisms, gene–environmental interactions. The terms were used combined and alone and both as MeSH terms and text words. In total, 57.755 articles were found. This number was reduced to 2588 by combining with colorectal neoplasm (MeSH Major topic) AND diet. The titles were evaluated and all prospective studies were sought identified. For food items, where no prospective studies were found, large case–control studies were sought retrieved. References, citations and related articles to found articles were scrutinised.

### Statistical analysis

Crude meta-analyses were conducted to assess potential interactions between NAT1 and NAT2 phenotypes and meat intake in relation to CRC risk by logistic regression analyses having both main and interaction effects and taking the potential effects of the studies into account. Predicted risks for each study were combined into a weighted average using the number of patients in the respective studies and odds ratios were calculated for each combination of meat intake and phenotype. The uncertainties and 95% confidence intervals were assessed by a bootstrap approach in which odds ratios were calculated from each of 8000 bootstrap samples.^7^ The binomial error was accommodated by a binomial resampling of the number of case from the total number of case and controls within each combination of meat intake and polymorphism.

## Results

Supplemental Tables S1 and S2 show the results from prospective, population-based studies and selected case–control studies on interactions between diet and susceptibility gene polymorphisms in relation to colorectal cancer.

### Meat

World Cancer Research Fund has concluded that there is convincing evidence that red (beef, pork, lamb, and goat) and processed meat are risk factors for CRC.^2007^ However, a recent analysis of prospective epidemiological studies concluded that the associations between CRC and red meat and processed meat are weak.^8^,^9^, The possible carcinogenic mechanisms underlying red and processed meat have recently been reviewed.^10^,^11^

**Figure 2:**
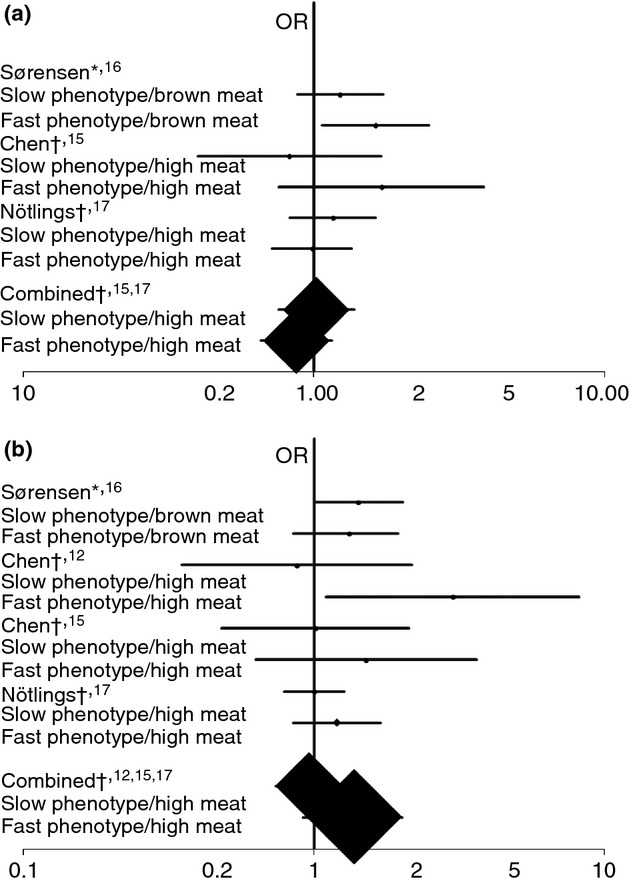
Odds ratios (OR) and 95% confidence interval (95% CI) for risk of CRC from prospective studies on *NAT1* (2a) or *NAT2* (2b) phenotypes respectively in the subgroups with high intake of meat or with preference for brown meat. *Odds ratio for slow and fast acetylators preferring brown to dark meat (reference: light to light brown meat). †Reference: in two studies^12^,^15^, fast and slow acetylators with high meat intake were compared to carriers of the same phenotype with low meat intake; in Ref. 17 all groups were compared to slow acetylators with low meat intake.

Red and processed meat represent sources of carcinogenic heterocyclic amines (HCA), polycyclic aromatic hydrocarbons (PAH) as well as N-nitroso compounds caused by cooking at high temperature and by processing of meat.^11^ N-acetyltransferases (coded for by *NAT1* and *NAT2*) are enzymes critical in the activation of HCA and PAH. Possible interaction between *NAT1* and *NAT2* polymorphisms and meat intake have been studied in prospective cohorts.^12^–^17^ Figure 2 shows the joint effects of high meat exposure and NAT phenotypes. Risk estimates for CRC for carriers of the fast or slow phenotypes for *NAT1* (Figure 2a) and *NAT2* (Figure 2b) in subgroups of high intake of meat or preference for brown meat. Meat intake was either quantified as number of servings per day or as total meat intake per day in grams. We made crude meta-analyses of the interaction between NAT1 and NAT2 phenotypes (slow or fast phenotype), respectively, and meat intake (low, medium or high) (Table 1 and Supplemental Table S3). The meta-analyses were based on studies where information on the number of participants in each group was available; *NAT1*,^15^,^17^ NAT2.^12^,^15^,^17^ There was no interaction between NAT1 phenotypes and meat intake in relation to risk of CRC (*P*-interaction 0.95) (Table 1). For *NAT2*, there was a tendency towards interaction between meat intake and NAT2 phenotypes (Table 1). Among carriers of the slow acetylator phenotype, risk of CRC was not affected by meat intake. Among carriers of the fast acetylator phenotype, low and medium meat intakes were not associated with risk of CRC (OR = 0.92, 95% CI: 0.68–1.20 and OR = 1.00, 95% CI: 0.69–1.45 respectively), whereas high meat intake was associated with higher risk of CRC (OR = 1.25, 95% CI: 0.92–2.01, *P*-interaction 0.07). High intake of meat was defined as 22.5–102.7 g meat per day,^17^ more than 1 serving per day,^15^ or more than 0.5 servings per day.^12^ In the prospective Diet, Health and Cancer cohort, the participants had a high mean daily meat intake of 167 g (5–95% percentiles 87–323 g per day) and a significantly higher risk among fast NAT1 acetylators who preferred brown to dark meat (OR 1.63, 95% CI: 1.07–2.49) compared with those who preferred light to light brown meat (reference group).^16^ Therefore, it cannot be excluded that genetically determined variation in *NAT* activity might be relevant for certain subgroups with high meat intake.

**Table 1:** Meta-analyses of prospective studies on interaction between *NAT1* or *NAT2* phenotypes respectively and meat intake in relation to risk of colorectal cancer

	Low meat intake OR (95% CI)	Medium meat intake OR (95% CI)	High meat intake OR (95% CI)	*P*-value‡
NAT 1*				
Slow acetylator	1 (ref)	.92 (0.70–1.23)	1.02 (0.76–1.38)	
Fast acetylator	.87 (0.66–1.14)	.82 (0.63–1.07)	.87 (0.66–1.15)	.95
NAT 2†				
Slow acetylator	1 (ref)	.99 (0.77–1.29)	.96 (0.74–1.24)	
Fast acetylator	.92 (0.68–1.20)	1.00 (0.69–1.45)	1.25 (0.92–2.01)	.07

The meta-analyses (Supplemental Table S3) were based on studies where information on the number of participants in each group was available.

* *NAT1*^15^,^17^

† *NAT2*.^12^,^15^,^17^ Odds ratios (OR), 95% confidence interval (95% CI).

‡ *P*-interaction. *P*-interactions were estimated by logistic regression analysis. Confidence intervals were obtained by bootstrapping.

ATP-binding cassette (ABC) transporters are abundant in the intestine and transport fatty acids, bacterial products, and dietary carcinogens, all of which may affect carcinogenesis. A significant interaction between genetic variation in *ABCB1*, but not *ABCC2* or *ABCG2*, and meat intake in relation to CRC was found.^6^,^18^, Homozygous *ABCB1* C3435T C-allele carriers were at 8% increased risk pr 25 g meat per day (95% CI: 1.00–1.16, *P*-interaction 0.02), whereas variant allele carriers were not at increased risk by meat intake. Fung *et al*. found that the *ABCB1* C3435T substitution changed the folding of the gene product due to ribosome stalling, thereby causing changes in substrate specificity.2009 Our experiments did not suggest any biological interaction between meat constituents (various preparations separated into both their water and lipid soluble phase) and ABCB1, either at the ATPase level of the protein, or at the transport level (T Litman *et al*., Copenhagen University, unpublished results). Thus, direct transport of meat carcinogens by ABCB1 does not seem to contribute to CRC.

Heme and iron in meat are considered potentially carcinogenic. Heme oxygenase-1 (HO-1) is the rate-limiting enzyme in the degradation of heme. Assessing the effect of functional polymorphisms in *HMOX1*, encoding HO-1, could determine whether heme or iron are major contributors to CRC. No interaction between meat intake and *HMOX1* A-413T was found in relation to risk of CRC in a prospective case–cohort study of 383 CRC cases and 763 randomly selected participants.^20^ This suggests that heme from meat is not important in CRC development.

Intestinal inflammation is a risk factor for CRC. A significant difference in the risk of CRC in relation to meat intake was observed among carriers of the *NFKB1* del-allele compared with homozygous carriers of the ins-allele.^21^ Carriers of *NFKB1* -94 del-allele were at 3% increased risk pr 25 g meat per day (95% CI: 0.98–1.09), whereas homozygous carriers of the ins-allele were not at increased risk (*P*-interaction 0.03). The *NFKB1* -94 del-allele leads to less promoter activity than the ins-allele, resulting in reduced p50 subunit synthesis.^22^ The NFκB p 50 homodimer seems to be specifically involved in anti-inflammatory effects in contrast to the pro-inflammatory p65/p50 unit. Hence, the results indicated that individuals with a low anti-inflammatory response increased their risk of developing CRC by meat intake in contrast to individuals with the homozygous ins-allele whose risk was unchanged by meat intake.^21^

O6-Methyl-guanine-DNA methyltransferase (MGTM) repairs DNA damage caused by alkylating agents including N-nitroso compounds from meat. A prospective study found that carriers of the *MGMT* Ile143Val variant were at 43% higher risk of CRC by a daily intake of meat above 56 g per day compared with no increased risk by high meat intake among homozygous wildtype carriers (*P*-interaction 0.04).^23^ This result suggests that individuals with low DNA repair capacity of oxidative DNA damage are at a high risk of CRC by meat intake in contrast to individuals with the homozygous wildtype, whose risk seems to be unchanged by meat intake.^23^

### Fish

High intake of n-3 PUFA from fish is considered to reduce inflammation-driven carcinogenesis in the colon. The anti-inflammatory effect of n-3 polyunsaturated fatty acids (PUFA) is mediated via the arachidonic acid pathway and leads to down-regulation of PGE2 and cyclo-oxygenase-2 (COX-2).^24^ Siezen and colleges analysed the possible interaction between fish intake and 23 polymorphisms in the arachidonic acid pathway in a prospective cohort including 160 cases and 367 controls.2006 No significant interactions were found.

### Fruit and vegetables

The chemopreventive effect of cruciferous vegetables is considered to be due to their high content of glucosinolate and glucosinolate metabolites, which are thought to cause apoptosis, inhibit cell proliferation and inhibit pro-inflammatory reactions by repressing NFκB. *GSTM1, GSTT1* and *GSTP1* encode the glucosinolates metabolising enzymes glutatione S-transferases. In the prospective population-based Singapore Chinese Health Study including 231 incident CRC cases and 1194 controls, a risk reduction of 69% was found by high intake of isothiocyanates among homozygous *GSTM1* and *GSTT1* null allele carriers vs. no risk reduction among wildtype allele carriers.^26^ These results could indicate that isothiocyanates from cruciferous vegetables protect against CRC in individuals with low GST activity.^26^

### Total energy, protein, fat, carbohydrate, milk, cereal and dairy product

Prospective studies found no significant associations between milk, cereal or dairy and *IL10* and *ABCC2* gene polymorphisms.^5^,^18^, No significant interactions between *PPARG* Pro12Ala and C161T polymorphisms and intake of fat, saturated fatty acids, monounsaturated fatty acids, n-6 PUFA, n-3 PUFA, or cholesterol in relation to risk of CRC were found in a case–control study.^27^

### Fibre

The protective effect of dietary fibre on risk of CRC is well documented.^28^ Fibre is the indigestible portion of plant foods whereof the insoluble fibre has bulking action and the soluble fibre is fermented by colonic bacteria to short chain fatty acid, including butyrate, which has anti-inflammatory properties.^29^ IL-10 is an anti-inflammatory cytokine and IL10−/− mice develop intestinal inflammation. Interaction between *IL10* C-592A and intake of fibre was found (*P*-interaction 0.02). Carriers of the low-activity-associated *IL10* C-592A variant allele eating <17.0 g of fibre per day had a significantly higher risk of colorectal cancer compared with reference group. In contrast, carriers of the same variant allele with high fibre intake had no change in risk compared with the reference group (reference: carriers of the homozygous wildtype and eating <17.0 g/day). This suggests that the increased risk caused by carrying the *IL10* low-activity variant C-592-A-allele can be overcome by high fibre intake. Thus, high intake of fibre seems to protect against CRC among individuals with genetically determined low IL10 activity.

### Vitamins

Dietary antioxidants such as vitamin C, vitamin E and carotene may reduce DNA alkylation by acting as nitrosation inhibitors. O6-Methyl-guanine-DNA methyltransferase (MGTM) is the DNA repair enzyme responsible for the cellular defence against alkylation damage in human cells. Possible interactions of *MGTM* Ile143Val polymorphism and these antioxidants were assessed in the European Prospective Investigation into Cancer and Nutrition (EPIC)-Norfolk cohort.^23^ Low intake of vitamin C, vitamin E and carotene intake was associated with a 27%, 46% and 43% higher risk of CRC respectively among carriers of the variant genotype compared with homozygous wildtype carriers with low intake (reference) (*P*-interaction 0.12, 0.009 and 0.005 respectively). In contrast, participants with high intake of vitamin C, vitamin E and carotene were not at risk of CRC.^23^ These results suggest that individuals with genetically determined low DNA repair capacity are at a high risk of CRC by low intake of vitamin C, vitamin E and carotene.

High intake of calcium and vitamin D has been inversely related to colorectal cancer. The vitamin D receptor (VDR), a nuclear hormone receptor, is essential for the action of vitamin D and calcium. A functional *VDR* start codon polymorphism (Fok1) results in a less effective enzyme encoded by the f variant allele compared with the F wildtype allele [43]. This polymorphism was assessed in conjunction with D vitamin in a prospective Chinese cohort of 217 cases and 890 controls.^30^ Although not significant, risk of colorectal cancer tended to be high among homozygous *VDR* variant genotypes with low intake of calcium in contrast to no change in risk by high intake of calcium (reference: homozygous wildtype genotype) (*P*-interaction 0.07). Also, a case–control study assessed the possible interaction between dietary intake of calcium and VDR polymorphisms in 2306 CRC cases and 2749 controls.^31^ The *Bsm* 1 and poly A *VDR* polymorphisms were evaluated. A significant 40% reduction in risk of rectal cancer was observed for the low-activity-associated homozygous *VDR LL* or *bb* genotypes when calcium intake was high (OR: 0.56, 95% CI: 0.36–0.85) (reference: homozygous *VDR LL* or *bb* genotypes and low calcium intake), whereas the observed risk for the homozygous *SS* or *BB* genotypes did not depend on calcium intake (*P*-interaction 0.01). No significant interactions were found for colon cancer.^31^ Together, these two studies might suggest that a high intake of calcium can overcome the risk conferred by a deficient *VDR*.

### Alcohol

In a study of the Danish Diet, Cancer and Health cohort, interaction between *PPARG* Pro12Ala and alcohol intake was found. Carriers of the variant allele of *PPARG* Pro12Ala were at increased risk of alcohol-related colorectal cancer (IRR 1.22 pr.10 g alcohol/day, 95% CI: 1.07–1.39), whereas homozygous wildtype Pro-allele carriers were not.^32^ The underlying mechanism of action was recently elucidated.^33^ Hormone replacement therapy is a risk factor for breast cancer, but seems to be associated with lowered risk of colorectal cancer.^34^ PPARγ is a negative regulator of the blood level of female sex hormones (via aromatase activity). Alcohol, in turn, inactivates PPARγ, leading to increased blood levels of female sex hormones. The *PPARG* Pro12Ala amino acid substitution abolishes the alcohol-specific regulation of *PPARG* presumably by modifying the interaction with the cofactor PGC-1α.^33^ The interaction has not been reproduced for other CRC cohorts, but the finding is indirectly supported by the fact that interaction between *PPARG* Pro12Ala and alcohol intake was also found in relation to breast cancer.^33^,^35^,

### Diet and Lynch syndrome (hereditary nonpolyposis colorectal cancer)

Lynch syndrome is caused by germline defects in one of the DNA mismatch repair (MMR) genes. A Dutch case–control study of 145 cases and 103 tumour-free controls with known or suspected Lynch syndrome found an inverse association between fruit intake and CRC (OR: 0.4 for highest vs. lowest tertile, 95% CI: 0.2–0.9).^36^

### Diet and epigenetic control of gene expression

Epigenetic modifications (DNA methylation, histone modifications and noncoding RNAs) have a fundamental role in the regulation of gene expression. Dietary folate (from green leaves) is the main source of methyl group necessary for DNA synthesis and DNA methylation. Prospective studies found that low dietary intake of folate and high alcohol consumption were associated with promoter hypermethylation of tumour suppressor genes^37^ and genome-wide DNA hypomethylation in CRC tissue from 122 and 609 patients respectively.^38^ Such modulations are considered to lead to inactivation of tumour suppressor genes and activation of oncogenes. A causal relationship between folate, alcohol and methylation changes was supported by animal studies.^39^,^40^, A subset of CRC patients are characterised by methylation abnormalities in a large number of genes (CpG island methylator phenotype (CIMP)). *MTHFR* encodes the methylenetetrahydrofolate reductase, which enables the utilisation of methyl groups for DNA synthesis. A high risk of CIMP-CRC was observed among *MTHFR* A1298C variant allele carriers with low intake of folate and methionine and high alcohol intake (OR: 2.1, 95% CI: 1.3–3.4), which was in contrast to variant allele carriers with high intake of folate and methionine and low alcohol intake (OR: 1.0, 95% CI: 0.5–1.8) in a case–control study of 916 cases and 1972 controls (*P*-interaction 0.03) (reference: homozygous wildtype genotype).^41^ This result may suggest that the risk associated with carrying the *MTHFR* A1298C variant allele can be overcome by a high intake of folate and methionine and low alcohol intake.

A recent meta-analysis found inverse association between blood selenium level and CRC in men.^42^ Although no human studies on selenium and epigenetic in relation to CRC were found, a causal relationship may be suggested by animal and colon cancer cell studies finding that selenium deficiency leads to DNA methylation abnormalities and that selenium supplementation suppressed aberrant DNA methylation.^40^ However, genetic variations in genes encoding selenoproteins were not associated with CRC risk and blood selenium level in a prospective nested case–control study of 804 colorectal cancer cases and 805 matched controls^42^ and no interaction was found between selenium intake and genetic polymorphisms in selenoproteins in relation to risk of CRC in a large case–control study including 2309 CRC cases.^43^

## Discussion

Candidate gene analyses of functional genetic polymorphisms, i.e. with known functional effects on the gene product such as enzymatic activity, allow interpretation of negative results as well as positive.^20^ Hence, the analysis may exclude the involvement of a gene in a process.^20^ Genome-wide association studies (GWAS) may be hampered by lack of knowledge on the functionality of the identified loci, heterogeneity in exposure among populations and difficulties in assessing environmental exposure.^44^ Genetic variants in different genes having similar phenotypic effects may act in the same functional pathway. Therefore, methods for pathway analyses to increase harvest are under development.^45^,^46^, Such strategy will increase the fraction explained by the genetic heritability of various diseases.

Potential sources of errors in assessment of diet in observational studies results from (i) inaccurate diet assessment, (ii) the fact that changes in diet occur over time, while diet assessment is usually only assessed once and (iii) co-variation in diet with other CRC risk factors. Co-linearity between red meat intake and other potential risk factors such as Western lifestyle, high intake of refined sugars and alcohol, low intake of fibre, fruits, vegetables, low physical activity and high smoking prevalence have been emphasised and may hamper the interpretation of the results.^8^

Prospective studies have the advantage that information on diet and lifestyle factors is collected for all participants at enrolment, which minimises the risk of differential misclassification between cases and controls. In particular, recall bias regarding food intake may hamper case–control studies.

Also, the intake of the diet under investigation should be sufficiently distributed in the population to allow evaluation of various intakes. The Danish ‘Diet, health and cancer’ cohort seems to provide a platform for studying the potential carcinogenic effect of red and processed meat because many Danes have a high meat intake. The impact of the combined effects of diet and genetic susceptibility may thus be evaluated. For example, the *NFKB1* -94ins/del del-allele was associated with risk of CRC among Swedes [odds ratio (OR) 3.81, 95% confidence interval (95% CI) 2.17–18.43, *P* < 0.0001 for heterozygous del-carriers and OR: 4.65, 95% CI: 2.43–8.89, *P* < 0.0001 for homozygous del-carriers], whereas no association was found among Chinese.^47^ An explanation for this apparent discrepancy may be suggested by the interaction between meat and *NFKB1* -94ins/del polymorphism found in the Danish cohort.^21^ Among *NFKB1* -94ins/del variant allele carriers, risk of CRC depends on meat intake. Thus, for intakes below 91.4 g red meat per day (the lowest tertile), OR was 1.19 (95% CI: 0.75–1.91), 1.79 (95% CI: 1.06–2.74) for intakes between 91.4 and 130.2 g per day, and 1.44 (95% CI: 0.89–2.34) for intakes above 130.2 g red meat per day (the highest tertile) respectively.^21^ Hence, the difference between the findings in the Danish and Swedish on one hand and the Chinese on the other may be related to differences in meat intake. In most other prospective studies, the study members had meat intakes, which correspond to the lowest tertile in the above-mentioned Danish study, and gene–meat interactions may thus be below detection level.^8^ Therefore, to allow comparison between populations, risk should ideally be reported per unit of exposure, e.g. per 100 g meat intake per day. When these criteria are met, studies on diet–gene interactions may provide valuable insight into the biological mechanisms underlying the various dietary items in colorectal carcinogenesis.

During the last years, it has become increasingly apparent that sporadic CRC encompasses a heterogeneous complex of diseases.^48^

Hence, multiple diet–gene interactions may contribute to CRC risk. To conclude, prospective studies suggest that dietary meat interact with *NAT1*, *NAT2*, *ABCB1*, *NFKB1*, but not *HMOX1*, *ABCC2* or *ABCG2*, cruciferous vegetables with *GSTM1*, *GSTT1*, and *CCND1*, calcium with *VDR*, vitamin C, vitamin E and carotene with *MGTM*, fibre with *IL10*, alcohol with *PPARG*, folate and alcohol with DNA methylation in relation to CRC. These interactions have to be replicated in other prospective studies before conclusions regarding biological mechanisms can be drawn. Further progress will involve the combined efforts of epidemiologists, molecular geneticists, statisticians and clinicians. Studies of diet–gene interactions necessitate large and well-designed cohorts such as population-based cohorts, prospectively recorded data, sufficient distribution of the intake of the examined diet in the cohort, the analyses of genetic variations in functional pathways, and, moreover, the comparison of cohorts with similar exposures. Such studies may provide valuable insight into the biological mechanisms underlying the various dietary items in colorectal carcinogenesis with the goal of reducing the prevalence of CRC.

## Authorship

*Guarantor of the article*: V. Andersen.

*Author contributions*: V. Andersen collected the material and wrote the manuscript. UV and VA discussed and revised the manuscript. RH did the statistical analysis. All authors approved the final version of the manuscript.

## Supporting Information

Table S1 Results from prospective, population-based studies on diet-gene interactions in relation to colorectal cancer.

Table S2 Table S2. Selected results from case-control studies on diet-susceptibility gene polymorphisms in relation to colorectal cancer.

Table S3 Crude meta-analyses of interaction between NAT1 and NAT2 phenotypes and meat intake in relation to risk of CRC in prospective studies. The meta-analyses were based on studies where information on the number of participants in each group was available, *NAT1*; (1;2) and *NAT2;*(1-3). Main and interaction effects of meat intake and NAT phenotype on risk of CRC estimated jointly from prospective studies.
