# Supplementary material for: Systematic review: diet–gene interactions and the risk of colorectal cancer
Source: Aliment Pharmacol Ther. 2012 Dec 10;37(4):383–91. doi: 10.1111/apt.12180 (PMC3565452; doi:10.1111/apt.12180)
Supplement: Table S1 — Results from prospective, population-based studies on diet-gene interactions in relation to colorectal cancer. [file apt0037-0383-sd1.docx]

**TABLE S1**. Results from prospective, population-based studies on diet-gene interactions in relation to colorectal cancer.

| Diet | Ethnicity | N_cases_ | N_cont_ | Gene | SNP/haplotype | rs number | *P* | comm | First  author | Year | Ref |
| --- | --- | --- | --- | --- | --- | --- | --- | --- | --- | --- | --- |
| *Meat* | | | | | | | | | | | |
| red and processed meat | Caucasian | 378 | 756 | NR1I2  (PXR) | A-24381C | rs1523127 | 0.28 |  | Andersen | 2010 | (1) |
| red and processed meat | Caucasian | 378 | 756 | NR1I2  (PXR) | C8055T | rs2276707 | 0.53 |  | Andersen | 2010 | (1) |
| red and processed meat | Caucasian | 378 | 756 | NR1I2  (PXR) | A7635G | rs6785049 | 0.72 |  | Andersen | 2010 | (1) |
| red and processed meat | Caucasian | 378 | 756 | NR1I2  (PXR) | C-rs1405655T | rs1405655 | 0.91 |  | Andersen | 2010 | (1) |
| red and processed meat | Caucasian | 378 | 756 | NR1I2  (PXR) | T-rs2695121C | rs2695121 | 1.28 |  | Andersen | 2010 | (1) |
| red and processed meat | Caucasian | 389 | 788 | ABCC2 | C-24T | rs717620 | 0.72 |  | Andersen | 2012 | (2) |
| red and processed meat | Caucasian | 389 | 788 | ABCC2 | G1249A | rs2273697 | 0.10 |  | Andersen | 2012 | (2) |
| red and processed meat | Caucasian | 389 | 788 | ABCC2 | C3972T | rs3740066 | 0.69 |  | Andersen | 2012 | (2) |
| red and processed meat | Caucasian | 359 | 765 | ABCB1 | C3435T | rs1045642 | 0.02 |  | Andersen | 2009 | (3) |
| red and processed meat | Caucasian | 359 | 765 | ABCB1 | G-rs3789243-A | G-rs3789243-A | 0.01 |  | Andersen | 2009 | (3) |
| red and processed meat | Caucasian | 359 | 765 | ABCG2 | C421A | rs2231142 | 0.40 |  | Andersen | 2009 | (3) |
| red and processed meat | Caucasian | 378 | 775 | IL10 | C-592A | rs1800872 | 0.92 |  | Andersen | 2012 | (4) |
| red and processed meat | Caucasian | 378 | 775 | IL10 |  | rs3024505 | 0.78 |  | Andersen | 2012 | (4) |
| red and processed meat | Caucasian | 383 | 763 | HMOX1 | A-413T | rs2071746 | 0.55 |  | Andersen | 2010 | (5) |
| meat | na | 183 | 443 | NAT2 | slow/rapid |  | 0.07 | F | Chan | 2005 | (6) |
| meat | Caucasian-American | 212 | 221 | NAT1 | slow/rapid |  | ns | M | Chen | 1998 | (7) |
| meat | Caucasian-American | 212 | 221 | NAT2 | slow/rapid |  | ns | M | Chen | 1998 | (7) |
| meat | Caucasian-American | 212 | 221 | NAT1/NAT2 | slow/rapid |  | ns | M | Chen | 1998 | (7) |
| red and processed meat | na (Norfolk, UK) | 273 | 2984 | MGMT | Ile143Val | rs2308321 | 0.04 | 6 | Loh | 2010 | (8) |
| red meat | Multiethnic | 1,009 | 1,522 | NAT2 | slow/intermediate vs rapid |  | 0.44 | 1 | Nöthlings | 2009 | (9) |
| processed meat | Multiethnic | 1,009 | 1,522 | NAT2 | slow/intermediate vs rapid |  | 0.13 | 1 | Nöthlings | 2009 | (9) |
| donenes | Multiethnic | 1,009 | 1,522 | NAT2 | slow/intermediate vs rapid |  | 0.42 | 1 | Nöthlings | 2009 | (9) |
| total HCA | Multiethnic | 398 | 1,444 | NAT2 | slow/intermediate vs rapid |  | 0.97 | 1 | Nöthlings | 2009 | (9) |
| DiMeIQx | Multiethnic | 398 | 1,444 | NAT2 | slow/intermediate vs rapid |  | 0.93 | 1 | Nöthlings | 2009 | (9) |
| PhIP | Multiethnic | 398 | 1,444 | NAT2 | slow/intermediate vs rapid |  | 0.49 | 1 | Nöthlings | 2009 | (9) |
| total meat | Caucasian | 379 | 769 | NAT1 | slow/rapid |  | ns | 2 | Sørensen | 2008 | (10) |
| fried meat | Caucasian | 379 | 769 | NAT1 | slow/rapid |  | ns | 2 | Sørensen | 2008 | (10) |
| processed meat | Caucasian | 379 | 769 | NAT1 | slow/rapid |  | ns | 2 | Sørensen | 2008 | (10) |
| red meat | Caucasian | 379 | 769 | NAT1 | slow/rapid |  | ns | 2 | Sørensen | 2008 | (10) |
| colour of meat surface  after pan-frying | Caucasian | 379 | 769 | NAT1 | slow/rapid |  |  | 2, 4 | Sørensen | 2008 | (10) |
| total meat | Caucasian | 379 | 769 | NAT2 | slow/rapid |  | ns | 3 | Sørensen | 2008 | (10) |
| fried meat | Caucasian | 379 | 769 | NAT2 | slow/rapid |  | ns | 3 | Sørensen | 2008 | (10) |
| processed meat | Caucasian | 379 | 769 | NAT2 | slow/rapid |  | ns | 3 | Sørensen | 2008 | (10) |
| red meat | Caucasian | 379 | 769 | NAT2 | slow/rapid |  | ns | 3 | Sørensen | 2008 | (10) |
| colour of meat surface  after pan-frying | Caucasian | 379 | 769 | NAT2 | slow/rapid |  |  | 3, 5 | Sørensen | 2008 | (10) |
| meat | Caucasian | 102 | 537 | NAT1 | slow/rapid |  | ns |  | Tiemersma | 2002 | (11) |
| poultry | Caucasian | 102 | 537 | NAT1 | slow/rapid |  | ns |  | Tiemersma | 2002 | (11) |
| meat | Caucasian | 102 | 537 | NAT2 | slow/rapid |  | ns |  | Tiemersma | 2002 | (11) |
| poultry | Caucasian | 102 | 537 | NAT2 | slow/rapid |  | ns |  | Tiemersma | 2002 | (11) |
| meat | Caucasian | 102 | 537 | GSTM1 | present/null |  | ns |  | Tiemersma | 2002 | (11) |
| poultry | Caucasian | 102 | 537 | GSTM1 | present/null |  | ns |  | Tiemersma | 2002 | (11) |
| *Fish* | | | | | | | | | | | |
| fish | Caucasian | 389 | 788 | ABCC2 | C-24T | rs717620 | 0.39 |  | Andersen | 2012 | (2) |
| fish | Caucasian | 389 | 788 | ABCC2 | G1249A | rs2273697 | 0.34 |  | Andersen | 2012 | (2) |
| fish | Caucasian | 389 | 788 | ABCC2 | C3972T | rs3740066 | 0.77 |  | Andersen | 2012 | (2) |
| fish | Caucasian | 378 | 775 | IL10 | C-592A | rs1800872 | 0.09 |  | Andersen | 2012 | (4) |
| fish | Caucasian | 378 | 775 | IL10 |  | rs3024505 | 0.51 |  | Andersen | 2012 | (4) |
| fish | na (Dutch) | 160 | 397 | PPARS | C789T |  | ns |  | Siezen | 2006 | (12) |
| fish | na (Dutch) | 160 | 397 | PPARS |  | rs2076167 | ns |  | Siezen | 2006 | (12) |
| fish | na (Dutch) | 160 | 397 | PPARS |  | rs3734254 | ns |  | Siezen | 2006 | (12) |
| fish | na (Dutch) | 160 | 397 | PPARS |  | rs1053046 | ns |  | Siezen | 2006 | (12) |
| fish | na (Dutch) | 160 | 397 | PPARS |  | rs9794 | ns |  | Siezen | 2006 | (12) |
| fish | na (Dutch) | 160 | 397 | PTGS1 |  | rs1236913 | ns |  | Siezen | 2006 | (12) |
| fish | na (Dutch) | 160 | 397 | PTGS1 |  | rs5789 | ns |  | Siezen | 2006 | (12) |
| fish | na (Dutch) | 160 | 397 | PLA2G4A |  | rs2307200 | ns |  | Siezen | 2006 | (12) |
| fish | na (Dutch) | 160 | 397 | PLA2G4A |  | rs6661772 | ns |  | Siezen | 2006 | (12) |
| fish | na (Dutch) | 160 | 397 | PLA2G4A |  | rs2307198 | ns |  | Siezen | 2006 | (12) |
| fish | na (Dutch) | 160 | 397 | PLA2G4A |  | rs12720707 | ns |  | Siezen | 2006 | (12) |
| fish | na (Dutch) | 160 | 397 | PLA2G2A |  | rs11573156 | ns |  | Siezen | 2006 | (12) |
| fish | na (Dutch) | 160 | 397 | PLA2G2A |  | rs2236771 | ns |  | Siezen | 2006 | (12) |
| fish | na (Dutch) | 160 | 397 | PLA2G2A |  | rs11677 | ns |  | Siezen | 2006 | (12) |
| fish | na (Dutch) | 160 | 397 | ALOX15 |  | rs2664592 | ns |  | Siezen | 2006 | (12) |
| fish | na (Dutch) | 160 | 397 | ALOX15 |  | rs11568141 | ns |  | Siezen | 2006 | (12) |
| fish | na (Dutch) | 160 | 397 | ALOX15 |  | rs743646 | ns |  | Siezen | 2006 | (12) |
| fish | Caucasian | 102 | 537 | NAT1 | slow/rapid |  | ns |  | Tiemersma | 2002 | (11) |
| fish | Caucasian | 102 | 537 | NAT2 | slow/rapid |  | ns |  | Tiemersma | 2002 | (11) |
| fish | Caucasian | 102 | 537 | GSTM1 | present/null |  | ns |  | Tiemersma | 2002 | (11) |
| *Fruit & vegetables* | | | | | | | | | | | |
| cruciferous vegetables | Chinese | 213 | 1194 | GSTM1 | Null-null vs null | na | ns |  | Seow | 2002 | (13) |
| cruciferous vegetables | Chinese | 213 | 1194 | GSTT1 | Null-null vs null | na | ns |  | Seow | 2002 | (13) |
| cruciferous vegetables | Chinese | 213 | 1194 | GSTP1 | AA vs AB and BB | na | ns |  | Seow | 2002 | (13) |
| cruciferous vegetables | Chinese | 213 | 1194 | GSTM1/  GSTT1 | combined | na |  | 8 | Seow | 2002 | (13) |
| *Cereal products* | | | | | | | | | | | |
| cereal products | Caucasian | 378 | 775 | IL10 | C-592A | rs1800872 | 0.63 |  | Andersen | 2012 | (4) |
| cereal products | Caucasian | 378 | 775 | IL10 |  | rs3024505 | 0.09 |  | Andersen | 2012 | (4) |
| cereal products | Caucasian | 389 | 788 | ABCC2 | C-24T | rs717620 | 0.50 |  | Andersen | 2012 | (2) |
| cereal products | Caucasian | 389 | 788 | ABCC2 | G1249A | rs2273697 | 0.50 |  | Andersen | 2012 | (2) |
| cereal products | Caucasian | 389 | 788 | ABCC2 | C3972T | rs3740066 | 0.49 |  | Andersen | 2012 | (2) |
| *Fibers* | | | | | | | | | | | |
| fibers | Caucasian | 389 | 788 | ABCC2 | C-24T | rs717620 | 0.07 |  | Andersen | 2012 | (2) |
| fibers | Caucasian | 389 | 788 | ABCC2 | G1249A | rs2273697 | 0.57 |  | Andersen | 2012 | (2) |
| fibers | Caucasian | 389 | 788 | ABCC2 | C3972T | rs3740066 | 0.45 |  | Andersen | 2012 | (2) |
| fiber | Caucasian | 378 | 775 | IL10 | C-592A | rs1800872 | 0.15 |  | Andersen | 2012 | (4) |
| fiber | Caucasian | 378 | 775 | IL10 |  | rs3024505 | 0.01 |  | Andersen | 2012 | (4) |
| *Total energy, fat, a.s.o.* | | | | | | | | | | | |
| meat and fat pattern1 | Multiethnic | 1,009 | 1,522 | NAT2 | slow/intermediate vs rapid |  | 0.04 | 1,7 | Nöthlings | 2009 | (9) |
| fat | Chinese | 217 | 890 | VDR | start codon polymorphism (FokI) |  | 0.19 |  | Wong | 2003 | (14) |
| *Vitamins* | | | | | | | | | | | |
| Vitamin E | na (Norfolk, UK) | 273 | 2984 | MGMT | Ile143Val | rs2308321 | 0.009 | 9 | Loh | 2010 | (8) |
| Carotene | na (Norfolk, UK) | 273 | 2984 | MGMT | Ile143Val | rs2308321 | 0.005 | 10 | Loh | 2010 | (8) |
| calcium | Chinese | 217 | 890 | VDR | start codon polymorphism (FokI) |  | 0.07 |  | Wong | 2003 | (14) |
| *Alcohol* | | | | | | | | | | | |
| Alcohol | Caucasian | 355 | 753 | PPARG | Pro12Ala | rs1801282 | 0.02 |  | Vogel | 2007 | (15) |

*P*-interaction

^1^A cooked meat module (inclusive PhIP, MeIQx, and DiMeIQx) was assessed approximately 5 years after baseline.

^2^Slow/rapid acetylator based on G445A (rs4987076), T1088A (rs1057126), C1095A (rs15561)

^3^Slow/rapid acetylator based on C481T (rs179929), G590A (rs1799930), A830G (rs1208), G857A (rs1799931)

^4^Odds ratio (OR) 1.63 (95% confidence interval (CI): 1.07-2.49) for NAT1 fast acetylators preferring brown to dark meat after pan-frying (reference: NAT1 fast acetylators preferring light to light brown meat after pan-frying)

^5^OR 1.42 (95% CI: 1.00-2.02) for NAT2 slow acetylators preferring brown to dark meat after pan-frying (reference: NAT2 slow acetylators preferring light to light brown meat after pan-frying)

^6^Variant allele carriers were at higher risk by high meat intake that homozygous wildtype allele carriers

^7^High risk among NAT2 rapid acetylators and high meat and fat intake compared to slow NAT2 acetylators and low meat and fat intake (OR 1.50 (95% CI: 1.08-2.10, *P-interaction* 0.047))

^8^A 57% (OR 0.43, 95% CI 0.20-0.96) lower risk for combined variants (low GST activity) and high dietary intake of isothiocyanates from cruciferous vegetables (reference group: combined variants and low dietary intake).

^9^Variant allele carriers were at higher risk by low Vitamine E intake that homozygous wildtype allele carriers

^10^Variant allele carriers were at higher risk by low carotene intake that homozygous wildtype allele carriers

HCA, heterocyclic amine; PhIP, 2-amino-1-methyl-6-phenylimidazo[4,5-*b*]pyridine; MeIQx, 2-amino-3,8-dimethylimidazo[4,5-*f*]quinoxaline; DiMeIQx, 2-amino-3,4,8-trimethylimidazo[4,5-*f*]quinoxaline;

Reference List

(1) Andersen V, Christensen J, Overvad K, Tjonneland A, Vogel U. Polymorphisms in NFkB, PXR, LXR and risk of colorectal cancer in a prospective study of Danes. BMC Cancer 2010 Sep 13;10(1):484.

(2) Andersen V, Egeberg R, Tjonneland A, Vogel U. ABCC2 transporter gene polymorphisms, diet and risk of colorectal cancer: a Danish prospective cohort study. Scand J Gastroenterol 2012 May;47(5):572-4.

(3) Andersen V, Ostergaard M, Christensen J, Overvad K, Tjonneland A, Vogel U. Polymorphisms in the xenobiotic transporter Multidrug Resistance 1 (MDR1) gene and interaction with meat intake in relation to risk of colorectal cancer in a Danish prospective case-cohort study. BMC Cancer 2009 Nov 21;9(1):407.

(4) Andersen V, Egebjerg R, Tjonneland A, Vogel U. Interaction between interleukin-10 (IL-10) polymorphisms and dietary fibre in relation to risk of colorectal cancer in a Danish case-cohort study. BMC Cancer 2012 May 17;12(1):183.

(5) Andersen V, Christensen J, Overvad K, Tjonneland A, Vogel U. Heme oxygenase-1 (HO-1) polymorphism is not associated with risk of colorectal cancer; a Danish prospective study. Eur J Gastroenterol Hepatol 2010.

(6) Chan AT, Tranah GJ, Giovannucci EL, Willett WC, Hunter DJ, Fuchs CS. Prospective study of N-acetyltransferase-2 genotypes, meat intake, smoking and risk of colorectal cancer. Int J Cancer 2005 Jul 1;115(4):648-52.

(7) Chen J, Stampfer MJ, Hough HL, Garcia-Closas M, Willett WC, Hennekens CH, et al. A prospective study of N-acetyltransferase genotype, red meat intake, and risk of colorectal cancer. Cancer Res 1998 Aug 1;58(15):3307-11.

(8) Loh YH, Mitrou PN, Bowman R, Wood A, Jeffery H, Luben RN, et al. MGMT Ile143Val polymorphism, dietary factors and the risk of breast, colorectal and prostate cancer in the European Prospective Investigation into Cancer and Nutrition (EPIC)-Norfolk study. DNA Repair (Amst) 2010 Apr 4;9(4):421-8.

(9) Nothlings U, Yamamoto JF, Wilkens LR, Murphy SP, Park SY, Henderson BE, et al. Meat and heterocyclic amine intake, smoking, NAT1 and NAT2 polymorphisms, and colorectal cancer risk in the multiethnic cohort study. Cancer Epidemiol Biomarkers Prev 2009 Jul;18(7):2098-106.

(10) Sorensen M, Autrup H, Olsen A, Tjonneland A, Overvad K, Raaschou-Nielsen O. Prospective study of NAT1 and NAT2 polymorphisms, tobacco smoking and meat consumption and risk of colorectal cancer. Cancer Lett 2008 Aug 8;266(2):186-93.

(11) Tiemersma EW, Kampman E, Bueno de Mesquita HB, Bunschoten A, van Schothorst EM, Kok FJ, et al. Meat consumption, cigarette smoking, and genetic susceptibility in the etiology of colorectal cancer: results from a Dutch prospective study. Cancer Causes Control 2002 May;13(4):383-93.

(12) Siezen CL, Bueno-de-Mesquita HB, Peeters PH, Kram NR, van DM, van Kranen HJ. Polymorphisms in the genes involved in the arachidonic acid-pathway, fish consumption and the risk of colorectal cancer. Int J Cancer 2006 Jul 15;119(2):297-303.

(13) Seow A, Yuan JM, Sun CL, van den BD, Lee HP, Yu MC. Dietary isothiocyanates, glutathione S-transferase polymorphisms and colorectal cancer risk in the Singapore Chinese Health Study. Carcinogenesis 2002 Dec;23(12):2055-61.

(14) Wong HL, Seow A, Arakawa K, Lee HP, Yu MC, Ingles SA. Vitamin D receptor start codon polymorphism and colorectal cancer risk: effect modification by dietary calcium and fat in Singapore Chinese. Carcinogenesis 2003 Jun;24(6):1091-5.

(15) Vogel U, Christensen J, Dybdahl M, Friis S, Hansen RD, Wallin H, et al. Prospective study of interaction between alcohol, NSAID use and polymorphisms in genes involved in the inflammatory response in relation to risk of colorectal cancer. Mutat Res 2007 Nov 1;624(1-2):88-100.
