# Supplementary material for: Systematic review: diet–gene interactions and the risk of colorectal cancer
Source: Aliment Pharmacol Ther. 2012 Dec 10;37(4):383–91. doi: 10.1111/apt.12180 (PMC3565452; doi:10.1111/apt.12180)
Supplement: Table S2 — Selected results from case-control studies on diet-susceptibility gene polymorphisms in relation to colorectal cancer. [file apt0037-0383-sd2.docx]

**TABLE S2.** Selected results from case-control studies on diet-susceptibility gene polymorphisms in relation to colorectal cancer.

| Diet | Ethnicity | Study design | N_cases_ | N_cont_ | Gene | SNP | rsnumber/  haplotype | *P* | com | First author | Year | Ref |
| --- | --- | --- | --- | --- | --- | --- | --- | --- | --- | --- | --- | --- |
| *Meat* | | | | | | | | | | | | |
| red meat | Hawai/Japan/Cauc | case-control | 521 | 639 | CYP2E1 | RsaI | na | 0.28 |  | Marchand | 2002 | (1) |
| red meat | Hawai/Japan/Cauc | case-control | 521 | 639 | CYP2E1 | Insert | na | 0.79 | 8 | Marchand | 2002 | (1) |
| red meat | Caucasian | case-control | 500 | 742 | GSTP | Ile105Val | na | 0.02 |  | Turner | 2004 | (2) |
| red meat | Caucasian | case-control | 500 | 742 | GSTP | Ala114Val | na | 0.27 |  | Turner | 2004 | (2) |
| red meat | Caucasian | case-control | 500 | 742 | EPHX1 | Tyr113H | na | 0.53 | 1 | Turner | 2004 | (2) |
| red meat | Caucasian | case-control | 500 | 742 | EPHX1 | His139Arg | na | 0.62 |  | Turner | 2004 | (2) |
| red meat | Caucasian | case-control | 500 | 742 | CYP1A1 | E7 | na | 0.88 |  | Turner | 2004 | (2) |
| red meat | Caucasian | case-control | 500 | 742 | CYP1A1 | Msp | na | 0.93 |  | Turner | 2004 | (2) |
| red meat | Caucasian | case-control | 500 | 742 | NQO1 | Pro187Ser | na | 0.32 |  | Turner | 2004 | (2) |
| red meat | Caucasian | case-control | 500 | 742 | NQO1 | Arg 139Trp | na | 0.14 |  | Turner | 2004 | (2) |
| meat | na (Taiwan) | case-control | 727 | 736 | GSTM1 | na | na | ns |  | Yeh | 2005 | (3) |
| meat | na (Taiwan) | case-control | 727 | 736 | GSTP1 | Ile105Val | na | ns |  | Yeh | 2005 | (3) |
| meat | na (Taiwan) | case-control | 727 | 736 | GSTT1 | na | na | ns |  | Yeh | 2005 | (3) |
| meat | na (Taiwan) | case-control | 727 | 736 | XRCC1 | Arg399Gln | na | 0.43 |  | Yeh | 2005 | (4) |
| meat | na (Taiwan) | case-control | 727 | 736 | XRCC3 | Thr241Met | na | 0.02 | 2 | Yeh | 2005 | (4) |
| meat | na (Taiwan) | case-control | 727 | 736 | XPD | Lys751Gln | na | 0.47 |  | Yeh | 2005 | (4) |
| red meat | Caucasian | case-control | 906 | 911 | APOE4 | Cys112Arg | rs429358 | *1 | 3 | Mrkonjic | 2009 | (5) |
| red meat | Caucasian | case-control | 906 | 911 | APOE2 | Arg158Cys | rs7412 | *1 |  | Mrkonjic | 2009 | (5) |
| meat | Caucasian | case-only | 1656 | 2292 | APC | Asp1822Val | | 0.11 |  | Theodoratou | 2008 | (6) |
| red meat | Caucasian | case-only | 1656 | 2292 | APC | Asp1822Val | | 0.002 | 4 | Theodoratou | 2008 | (6) |
| meat | Caucasian | case-only | 1656 | 2292 | APC | Glu1319Gln | | 0.84 |  | Theodoratou | 2008 | (6) |
| red meat | Caucasian | case-only | 1656 | 2292 | APC | Glu1319Gln | | 0.24 |  | Theodoratou | 2008 | (6) |
| meat | Japanese | case-control | 128 | 238 | PPAR | Pro12Ala | rs1801282 | 0.64 | 8 | Kuriki | 2006 | (7) |
| meat | Japanese | case-control | 128 | 238 | PPAR | C161T | rs3856806 | 0.21 |  | Kuriki | 2006 | (7) |
| beef and pork | Japanese | case-control | 257 | 771 | PPAR | Pro12Ala | rs1801282 | 0.55 |  | Kuriki | 2006 | (7) |
| beef and pork | Japanese | case-control | 257 | 771 | PPAR | C161T | rs3856806 | 0.69 |  | Kuriki | 2006 | (7) |
| processed meat | Japanese | case-control | 257 | 771 | PPAR | Pro12Ala | rs1801282 | 0.81 |  | Kuriki | 2006 | (7) |
| processed meat | Japanese | case-control | 257 | 771 | PPAR | C161T | rs3856806 | 0.15 |  | Kuriki | 2006 | (7) |
| poultry | Japanese | case-control | 257 | 771 | PPAR | Pro12Ala | rs1801282 | 0.25 |  | Kuriki | 2006 | (7) |
| poultry | Japanese | case-control | 257 | 771 | PPAR | C161T | rs3856806 | 0.46 |  | Kuriki | 2006 | (7) |
| *Fish* | | | | | | | | | | | | |
| fish | na (Taiwan) | case-control | 727 | 736 | XRCC1 | Arg399Gln | na | 0.21 |  | Yeh | 2005 | (4) |
| fish | na (Taiwan) | case-control | 727 | 736 | XRCC3 | Thr241Met | na | 0.98 |  | Yeh | 2005 | (4) |
| fish | na (Taiwan) | case-control | 727 | 736 | XPD | Lys751Gln | na | 0.86 |  | Yeh | 2005 | (4) |
| fish | Japanese | case-control | 257 | 771 | PPAR | Pro12Ala | rs1801282 | 0.49 |  | Kuriki | 2006 | (7) |
| fish | Japanese | case-control | 257 | 771 | PPAR | C161T | rs3856806 | 0.75 |  | Kuriki | 2006 | (7) |
| *Fruit & vegetables* | | | | | | | | | | | | |
| vegetable | Caucasian | case-control | 500 | 742 | GSTP | Ile105Val | na | 0.85 |  | Turner | 2004 | (2) |
| fruit | Caucasian | case-control | 500 | 742 | GSTP | Ile105Val | na | 0.23 |  | Turner | 2004 | (2) |
| vegetable | Caucasian | case-control | 500 | 742 | GSTP | Ala114Val | na | 0.69 |  | Turner | 2004 | (2) |
| fruit | Caucasian | case-control | 500 | 742 | GSTP | Ala114Val | na | 0.48 |  | Turner | 2004 | (2) |
| vegetable | Caucasian | case-control | 500 | 742 | EPHX1 | Tyr113H | na | 0.39 | 1 | Turner | 2004 | (2) |
| fruit | Caucasian | case-control | 500 | 742 | EPHX1 | Tyr113H | na | 0.03 | 1 | Turner | 2004 | (2) |
| vegetable | Caucasian | case-control | 500 | 742 | EPHX1 | His139Arg | na | 0.38 |  | Turner | 2004 | (2) |
| fruit | Caucasian | case-control | 500 | 742 | EPHX1 | His139Arg | na | 0.59 |  | Turner | 2004 | (2) |
| vegetable | Caucasian | case-control | 500 | 742 | CYP1A1 | E7 | na | 0.65 |  | Turner | 2004 | (2) |
| fruit | Caucasian | case-control | 500 | 742 | CYP1A1 | E7 | na | 0.93 |  | Turner | 2004 | (2) |
| vegetable | Caucasian | case-control | 500 | 742 | CYP1A1 | Msp | na | 0.22 |  | Turner | 2004 | (2) |
| fruit | Caucasian | case-control | 500 | 742 | CYP1A1 | Msp | na | 0.04 |  | Turner | 2004 | (2) |
| vegetable | Caucasian | case-control | 500 | 742 | NQO1 | Pro187Ser | na | 0.54 |  | Turner | 2004 | (2) |
| fruit | Caucasian | case-control | 500 | 742 | NQO1 | Pro187Ser | na | 0.98 |  | Turner | 2004 | (2) |
| vegetable | Caucasian | case-control | 500 | 742 | NQO1 | Arg 139Trp | na | 0.63 |  | Turner | 2004 | (2) |
| fruit | Caucasian | case-control | 500 | 742 | NQO1 | Arg 139Trp | na | 0.98 |  | Turner | 2004 | (2) |
| vegetable/fruit | na (Taiwan) | case-control | 727 | 736 | GSTT1 | Null vs present | na | 0.21 | M | Yeh | 2005 | (3) |
| vegetable/fruit | na (Taiwan) | case-control | 727 | 736 | GSTT1 | Null vs present | na | 0.06 | F | Yeh | 2005 | (3) |
| vegetable/fruit | na (Taiwan) | case-control | 727 | 736 | GSTP1 | AA/with G | na | 0.03 | M | Yeh | 2005 | (3) |
| vegetable/fruit | na (Taiwan) | case-control | 727 | 736 | GSTP1 | AA/with G | na | 0.42 | F | Yeh | 2005 | (3) |
| vegetable | na (Taiwan) | case-control | 727 | 736 | XRCC1 | Arg399Gln | na | 0.91 |  | Yeh | 2005 | (4) |
| vegetable | na (Taiwan) | case-control | 727 | 736 | XRCC3 | Thr241Met | na | 0.38 |  | Yeh | 2005 | (4) |
| vegetable | na (Taiwan) | case-control | 727 | 736 | XPD | Lys751Gln | na | 0.44 |  | Yeh | 2005 | (4) |
| *Diary products* | | | | | | | | | | | | |
| milk | Japanese | case-control | 128 | 238 | PPAR | Pro12Ala | rs1801282 | 0.08 |  | Kuriki | 2006 | (7) |
| milk | Japanese | case-control | 128 | 238 | PPAR | C161T | rs3856806 | 0.67 |  | Kuriki | 2006 | (7) |
| egg | Japanese | case-control | 257 | 771 | PPAR | Pro12Ala | rs1801282 | 0.07 |  | Kuriki | 2006 | (7) |
| egg | Japanese | case-control | 257 | 771 | PPAR | C161T | rs3856806 | 0.20 |  | Kuriki | 2006 | (7) |
| milk | Japanese | case-control | 257 | 771 | PPAR | Pro12Ala | rs1801282 | 0.39 |  | Kuriki | 2006 | (7) |
| milk | Japanese | case-control | 257 | 771 | PPAR | C161T | rs3856806 | 0.88 |  | Kuriki | 2006 | (7) |
| youghurt | Japanese | case-control | 257 | 771 | PPAR | Pro12Ala | rs1801282 | 0.70 |  | Kuriki | 2006 | (7) |
| youghurt | Japanese | case-control | 257 | 771 | PPAR | C161T | rs3856806 | 0.74 |  | Kuriki | 2006 | (7) |
| *Energy, fat, proteins, carbohydrates* | | | | | | | | | | | | |
| dietary cholesterol | Caucasian | case-control | 906 | 911 | APOE4 | Cys112Arg | rs429358 | *1 |  | Mrkonjic | 2009 | (5) |
| saturated fat | Caucasian | case-control | 906 | 911 | APOE4 | Cys112Arg | rs429358 | *1 |  | Mrkonjic | 2009 | (5) |
| total fat | Caucasian | case-control | 906 | 911 | APOE4 | Cys112Arg | rs429358 | *1 |  | Mrkonjic | 2009 | (5) |
| dietary cholesterol | Caucasian | case-control | 906 | 911 | APOE2 | Arg158Cys | rs7412 | *1 |  | Mrkonjic | 2009 | (5) |
| saturated fat | Caucasian | case-control | 906 | 911 | APOE2 | Arg158Cys | rs7412 | *1 |  | Mrkonjic | 2009 | (5) |
| total fat | Caucasian | case-control | 906 | 911 | APOE2 | Arg158Cys | rs7412 | *1 |  | Mrkonjic | 2009 | (5) |
| total fat | Caucasian | case-only | 1656 | 2292 | APC | Asp1822Val | | 0.27 |  | Theodoratou | 2008 | (6) |
| cholesterol | Caucasian | case-only | 1656 | 2292 | APC | Asp1822Val | | 0.51 |  | Theodoratou | 2008 | (6) |
| total FA | Caucasian | case-only | 1656 | 2292 | APC | Asp1822Val | | 0.34 |  | Theodoratou | 2008 | (6) |
| SFA | Caucasian | case-only | 1656 | 2292 | APC | Asp1822Val | | 0.61 |  | Theodoratou | 2008 | (6) |
| MUFA | Caucasian | case-only | 1656 | 2292 | APC | Asp1822Val | | 0.02 | 5 | Theodoratou | 2008 | (6) |
| PUFA | Caucasian | case-only | 1656 | 2292 | APC | Asp1822Val | | 0.86 |  | Theodoratou | 2008 | (6) |
| n6PUFA | Caucasian | case-only | 1656 | 2292 | APC | Asp1822Val | | 0.46 |  | Theodoratou | 2008 | (6) |
| n3PUFA | Caucasian | case-only | 1656 | 2292 | APC | Asp1822Val | | 0.09 |  | Theodoratou | 2008 | (6) |
| tFA | Caucasian | case-only | 1656 | 2292 | APC | Asp1822Val | | 0.86 |  | Theodoratou | 2008 | (6) |
| EPA | Caucasian | case-only | 1656 | 2292 | APC | Asp1822Val | | 0.02 | 6 | Theodoratou | 2008 | (6) |
| DHA | Caucasian | case-only | 1656 | 2292 | APC | Asp1822Val | | 0.01 | 7 | Theodoratou | 2008 | (6) |
| total fat | Caucasian | case-only | 1656 | 2292 | APC | Glu1319Gln | | 0.62 |  | Theodoratou | 2008 | (6) |
| cholesterol | Caucasian | case-only | 1656 | 2292 | APC | Glu1319Gln | | 0.13 |  | Theodoratou | 2008 | (6) |
| total FA | Caucasian | case-only | 1656 | 2292 | APC | Glu1319Gln | | 0.42 |  | Theodoratou | 2008 | (6) |
| SFA | Caucasian | case-only | 1656 | 2292 | APC | Glu1319Gln | | 0.58 |  | Theodoratou | 2008 | (6) |
| MUFA | Caucasian | case-only | 1656 | 2292 | APC | Glu1319Gln | | 0.09 |  | Theodoratou | 2008 | (6) |
| PUFA | Caucasian | case-only | 1656 | 2292 | APC | Glu1319Gln | | 0.57 |  | Theodoratou | 2008 | (6) |
| n6PUFA | Caucasian | case-only | 1656 | 2292 | APC | Glu1319Gln | | 0.43 |  | Theodoratou | 2008 | (6) |
| n3PUFA | Caucasian | case-only | 1656 | 2292 | APC | Glu1319Gln | | 0.90 |  | Theodoratou | 2008 | (6) |
| tFA | Caucasian | case-only | 1656 | 2292 | APC | Glu1319Gln | | 0.70 |  | Theodoratou | 2008 | (6) |
| mayonaise | Japanese | case-control | 257 | 771 | PPAR | Pro12Ala | rs1801282 | 0.62 |  | Kuriki | 2006 | (7) |
| mayonaise | Japanese | case-control | 257 | 771 | PPAR | C161T | rs3856806 | 0.23 |  | Kuriki | 2006 | (7) |
| fried foods | Japanese | case-control | 257 | 771 | PPAR | Pro12Ala | rs1801282 | 0.01 |  | Kuriki | 2006 | (7) |
| fried foods | Japanese | case-control | 257 | 771 | PPAR | C161T | rs3856806 | 0.86 |  | Kuriki | 2006 | (7) |
| deep-fried foods | Japanese | case-control | 257 | 771 | PPAR | Pro12Ala | rs1801282 | 0.17 |  | Kuriki | 2006 | (7) |
| deep-fried foods | Japanese | case-control | 257 | 771 | PPAR | C161T | rs3856806 | 0.43 |  | Kuriki | 2006 | (7) |
| fat | Japanese | case-control | 257 | 771 | PPAR | Pro12Ala | rs1801282 | 0.15 |  | Kuriki | 2006 | (7) |
| fat | Japanese | case-control | 257 | 771 | PPAR | C161T | rs3856806 | 0.58 |  | Kuriki | 2006 | (7) |
| SFA | Japanese | case-control | 257 | 771 | PPAR | Pro12Ala | rs1801282 | 0.51 |  | Kuriki | 2006 | (7) |
| SFA | Japanese | case-control | 257 | 771 | PPAR | C161T | rs3856806 | 0.64 |  | Kuriki | 2006 | (7) |
| MUFA | Japanese | case-control | 257 | 771 | PPAR | Pro12Ala | rs1801282 | 0.14 |  | Kuriki | 2006 | (7) |
| MUFA | Japanese | case-control | 257 | 771 | PPAR | C161T | rs3856806 | 0.94 |  | Kuriki | 2006 | (7) |
| n-6PUFA | Japanese | case-control | 257 | 771 | PPAR | Pro12Ala | rs1801282 | na |  | Kuriki | 2006 | (7) |
| n-6PUFA | Japanese | case-control | 257 | 771 | PPAR | C161T | rs3856806 | 0.72 |  | Kuriki | 2006 | (7) |
| n-3PUFA | Japanese | case-control | 257 | 771 | PPAR | Pro12Ala | rs1801282 | 0.50 |  | Kuriki | 2006 | (7) |
| n-3PUFA | Japanese | case-control | 257 | 771 | PPAR | C161T | rs3856806 | 0.91 |  | Kuriki | 2006 | (7) |
| cholesterol | Japanese | case-control | 257 | 771 | PPAR | Pro12Ala | rs1801282 | 0.52 |  | Kuriki | 2006 | (7) |
| cholesterol | Japanese | case-control | 257 | 771 | PPAR | C161T | rs3856806 | 0.37 |  | Kuriki | 2006 | (7) |
| low fat dairy products | na (USA) | case-control | 2306 | 2749 | VDR | intron 8/3'UTR | | 0.62/0.30 |  | Slattery | 2004 | (8) |
| *Vitamins* | | | | | | | | | | | | |
| calcium | na (USA) | case-control | 2306 | 2749 | VDR | intron 8/3'UTR | | 0.51/0.01 | 9 | Slattery | 2004 | (8) |
| Vitamin D | na (USA) | case-control | 2306 | 2749 | VDR | intron 8/3'UTR | | 0.26/0.11 |  | Slattery | 2004 | (8) |
| *Various* | | | | | | | | | | | | |
| BMI | Hawai/Japan/Cauc | case-control | 521 | 639 | CYP2E1 | RsaI | na | ns |  | Marchand | 2002 | (1) |
| BMI | Hawai/Japan/Cauc | case-control | 521 | 639 | CYP2E1 | Insert | na | ns |  | Marchand | 2002 | (1) |
| ratio meat/fruit  and veg | na (Norwegian) | case-control | 234 | 400 | GSTM1 | Ile195Val | na | ns |  | Skjelbred | 2007 | (9) |
| ratio meat/fruit  and veg | na (Norwegian) | case-control | 234 | 400 | EPHX1 | His139Arg | rs2234922 | ns |  | Skjelbred | 2007 | (9) |
| ratio meat/fruit  and veg | na (Norwegian) | case-control | 234 | 400 | EPHX1 | Tyr113His | rs1051740 | ns |  | Skjelbred | 2007 | (9) |

*P*-trend or *p*-interaction

^1^HWE not achieved

^2^XRCC3 241Thr/Thr genotype and low meat intake were at high risk of CRC

^3^data analysed separate for mismatch-repair functionel status (red meat diet appears to increase the risk of MMR-deficient CRC among APOE4 carriers (p=0.06) and MMR-proficient CRC among non-APOE4 carriers

^4^carriers of the homozygous variant genotype with low intake of red meat had low risk of CRC compared to carriers of heterozygous and wildtype genotypes

^5^carriers of the homozygous variant genotype with low intake of MUFA had low risk of CRC compared to carriers of heterozygous and wildtype genotypes

^6^carriers of the homozygous variant genotype with low intake of EPA had low risk of CRC compared to carriers of heterozygous and wildtype genotypes

^7^carriers of the homozygous variant genotype with low intake of DHA had low risk of CRC compared to carriers of heterozygous and wildtype genotypes

^8^rectal cancer

^9^data separate for colon and rectal cancer

Abbreviations: HCA, heterocyclic amine; FA, fatty acids; na, non applicable; tFA, trans fatty

acids;

Reference List

(1) Le ML, Donlon T, Seifried A, Wilkens LR. Red meat intake, CYP2E1 genetic polymorphisms, and colorectal cancer risk. Cancer Epidemiol Biomarkers Prev 2002 Oct;11(10 Pt 1):1019-24.

(2) Turner F, Smith G, Sachse C, Lightfoot T, Garner RC, Wolf CR, et al. Vegetable, fruit and meat consumption and potential risk modifying genes in relation to colorectal cancer. Int J Cancer 2004 Nov 1;112(2):259-64.

(3) Yeh CC, Hsieh LL, Tang R, Chang-Chieh CR, Sung FC. Vegetable/fruit, smoking, glutathione S-transferase polymorphisms and risk for colorectal cancer in Taiwan. World J Gastroenterol 2005 Mar 14;11(10):1473-80.

(4) Yeh CC, Hsieh LL, Tang R, Chang-Chieh CR, Sung FC. MS-920: DNA repair gene polymorphisms, diet and colorectal cancer risk in Taiwan. Cancer Lett 2005 Jun 28;224(2):279-88.

(5) Mrkonjic M, Chappell E, Pethe VV, Manno M, Daftary D, Greenwood CM, et al. Association of apolipoprotein E polymorphisms and dietary factors in colorectal cancer. Br J Cancer 2009 Jun 16;100(12):1966-74.

(6) Theodoratou E, Campbell H, Tenesa A, McNeill G, Cetnarskyj R, Barnetson RA, et al. Modification of the associations between lifestyle, dietary factors and colorectal cancer risk by APC variants. Carcinogenesis 2008 Sep;29(9):1774-80.

(7) Kuriki K, Hirose K, Matsuo K, Wakai K, Ito H, Kanemitsu Y, et al. Meat, milk, saturated fatty acids, the Pro12Ala and C161T polymorphisms of the PPARgamma gene and colorectal cancer risk in Japanese. Cancer Sci 2006 Nov;97(11):1226-35.

(8) Slattery ML, Neuhausen SL, Hoffman M, Caan B, Curtin K, Ma KN, et al. Dietary calcium, vitamin D, VDR genotypes and colorectal cancer. Int J Cancer 2004 Sep;%20;111(5):750-6.

(9) Skjelbred CF, Saebo M, Hjartaker A, Grotmol T, Hansteen IL, Tveit KM, et al. Meat, vegetables and genetic polymorphisms and the risk of colorectal carcinomas and adenomas. BMC Cancer 2007 Dec;%19;7:228.:228.
