# Supplementary material for: Systematic review: diet–gene interactions and the risk of colorectal cancer
Source: Aliment Pharmacol Ther. 2012 Dec 10;37(4):383–91. doi: 10.1111/apt.12180 (PMC3565452; doi:10.1111/apt.12180)
Supplement: Table S3 — Crude meta-analyses of interaction between NAT1 and NAT2 phenotypes and meat intake in relation to risk of CRC in prospective studies. The meta-analyses were based on studies where information on the number of participants in each group was available, NAT1; (1;2) and NAT2;(1-3). Main and interaction effects of meat intake and NAT phenotype on risk of CRC estimated jointly from prospective studies. [file apt0037-0383-sd3.docx]

**TABLE S3**. Crude meta-analyses of interaction between NAT1 and NAT2 phenotypes and meat intake in relation to risk of CRC in prospective studies. The meta-analyses were based on studies where information on the number of participants in each group was available, *NAT1*; (1;2) and *NAT2;*(1-3). Main and interaction effects of meat intake and NAT phenotype on risk of CRC estimated jointly from prospective studies.

| **Parameter** | **OR** | **Std. Error** | **p-value^1^** |
| --- | --- | --- | --- |
| NAT1 |  |  |  |
| Intercept | 1.054 | 0.1446 | 0.702 |
| Meat – medium intake versus low | 0.922 | 0.1340 | 0.577 |
| Meat – high intake versus low | 1.021 | 0.1560 | 0.894 |
| Enzyme – Fast versus slow phenotype | 0.866 | 0.1226 | 0.311 |
| Study – Nötlings (17) alone | 0.659 | 0.0715 | 0.0001 |
| Meat – medium intake: fast versus slow phenotype | 1.027 | 0.2002 | 0.890 |
| Meat – high: fast versus slow phenotype | 0.987 | 0.1200 | 0.950 |
| NAT2 |  |  |  |
| Intercept | 0.665 | 0.049 | <0.001 |
| Meat – medium intake versus low | 0.993 | 0.102 | 0.948 |
| Meat – high intake versus low | 0.964 | 0.101 | 0.725 |
| Enzyme – Fast versus slow phenotype | 0.922 | 0.107 | 0.485 |
| Study – Nötlings (17) alone | 1.439 | 0.155 | 0.001 |
| Study – Chan (12) alone | 0.636 | 0.069 | <0.001 |
| Meat – medium intake: fast versus slow phenotype | 1.089 | 0.202 | 0.650 |
| Meat – high: fast versus slow phenotype | 1.411 | 0.266 | 0.068 |

^1^P-value adjusted for study effects. Interactions between meat intake and *NAT1* and *NAT2* phenotypes in relation to risk of CRC were assessed by logistic regression analyses having both main and interaction effects. Studies were analysed combined and alone to test the robustness of the data. High intake of meat was defined as 22.5-102.7 g meat per day (1), more than 1 serving per day (2), or more than 0.5 servings per day (3).

Reference List

(1) Nothlings U, Yamamoto JF, Wilkens LR, Murphy SP, Park SY, Henderson BE, et al. Meat and heterocyclic amine intake, smoking, NAT1 and NAT2 polymorphisms, and colorectal cancer risk in the multiethnic cohort study. Cancer Epidemiol Biomarkers Prev 2009 Jul;18(7):2098-106.

(2) Chen J, Stampfer MJ, Hough HL, Garcia-Closas M, Willett WC, Hennekens CH, et al. A prospective study of N-acetyltransferase genotype, red meat intake, and risk of colorectal cancer. Cancer Res 1998 Aug 1;58(15):3307-11.

(3) Chan AT, Tranah GJ, Giovannucci EL, Willett WC, Hunter DJ, Fuchs CS. Prospective study of N-acetyltransferase-2 genotypes, meat intake, smoking and risk of colorectal cancer. Int J Cancer 2005 Jul 1;115(4):648-52.
